# Investigation on sexual function in young breast cancer patients during endocrine therapy: a latent class analysis

**DOI:** 10.3389/fmed.2023.1218369

**Published:** 2023-07-06

**Authors:** Lu Gan, Yi-Ming Miao, Xiao-Jing Dong, Qi-Rong Zhang, Qing Ren, Nan Zhang

**Affiliations:** ^1^Comprehensive Breast Health Center, Ruijin Hospital, Shanghai Jiao Tong University School of Medicine, Shanghai, China; ^2^School of Medicine, Shanghai Jiao Tong University, Shanghai, China

**Keywords:** breast cancer, postoperative, endocrine therapy, female sexual dysfunction, latent class analysis

## Abstract

**Backgrounds:**

The aim of this study was to investigate the sexual function status of young breast cancer patients during endocrine therapy, identify potential categories of sexual function status, and analyze the factors affecting the potential categories of sexual function status during endocrine therapy.

**Methods:**

A cross-sectional survey was conducted on 189 young breast cancer patients who underwent postoperative adjuvant endocrine therapy in Shanghai Ruijin Hospital. The latent class analysis was used to identify potential categories of patient sexual function characteristics with respect to the FSFI sex health measures. Logistic regression analysis was used to analyze the influencing factors for the high risk latent class groups. A nomogram prognostic model were then established to identify high risk patients for female sexual dysfunction (FSD), and C-index was used to determine the prognostic accuracy.

**Results:**

Patients were divided into a “high dysfunction-low satisfaction” group and a “low dysfunction-high satisfaction” group depending on the latent class analysis, accounting for 69.3% and 30.7%, respectively. Patients who received aromatase inhibitors (AI) combined with ovarian function suppression (OFS) treatment (*p* = 0.027), had poor body-image after surgery (*p* = 0.007), beared heavy medical economy burden(*p* < 0.001), and had a delayed recovery of sexual function after surgery (*p* = 0.001) were more likely to be classified into the “high dysfunction-low satisfaction” group, and then conducted into the nomogram. The *C*-index value of the nomogram for predicting FSD was 0.782.

**Conclusion:**

The study revealed the heterogeneity of sexual function status among young breast cancer patients during endocrine therapy, which may help identify high-risk patients and provide early intervention.

## Introduction

Breast cancer (BC) is currently the most common malignant tumor among women worldwide (1). The incidence of breast cancer in China is on a rapid upward trend, with a tendency toward younger patients (2). About 60% of patients are premenopausal at the time of diagnosis, while between 50% and 60% of premenopausal women with early-stage breast cancer are diagnosed as estrogen receptor-positive in China (3). Adjuvant endocrine therapy plays a vital role in reducing the risk of recurrence for these patients. However, endocrine therapy for breast cancer can lead to a decrease in hormone levels, which can cause sexual dysfunction, including sexual desire and arousal disorders, vaginal dryness, decreased tissue elasticity, and increased tissue fragility in the vulva and vagina, resulting in discomfort or pain during intercourse (4), thus triggering female sexual dysfunction.

Breast cancer patients may experience a range of physical changes resulting from comprehensive treatments, such as surgery, chemotherapy, radiotherapy, and targeted therapy. These changes include alterations in body image, hormonal fluctuations, and ovarian dysfunction, which may contribute to sexual dysfunction. Adjuvant endocrine therapy can cause or exacerbate menopausal symptoms in patients (5). Adding ovarian suppressants to classic endocrine therapy can bring therapeutic benefits, but can also increase toxic side effects, mainly manifested as menopausal symptoms, decreased sexual activity, and decreased quality of life (6). In addition to treatment factors, other factors such as age, mental health status, overall physical health, and marital relationships can also have a significant impact on sexual health and may be associated with sexual dysfunction (7).

Sexual quality is an important component of the quality of life of young cancer patients and an essential part of overall cancer management (8). Sexual activity is an important way for couples to express emotions and convey love. Sexual activity is a crucial means for couples to express emotions and convey affection, serving as both a lubricant for enhancing conjugal harmony and a vital way of maintaining a healthy marriage. During sexual intercourse, the body releases beta-endorphins, a natural sedative and analgesic that creates a relaxed and carefree internal environment in the nervous system, stabilizes emotions and behavior, and relieves symptoms such as anxiety and depression (9). High levels of sexual satisfaction can make people happy and improve their physical and mental well-being, stabilize their marital relationships, and enhance their immune function, which is conducive to disease recovery.

As endocrine therapy lasts for 5 years or even longer, it is meaningful to pay attention to the sexual function of breast cancer patients during adjuvant endocrine therapy. Younger patients are more concerned about their quality of life, especially their sexual quality of life, compared to middle-aged and older patients. In the cultural context of China, young patients often bear heavier social responsibilities and family burdens, and may face more difficulties. Currently, there is limited research focused on the sexual function of young breast cancer patients during endocrine therapy, so it is necessary to investigate and analyze the current status of sexual function and its related factors in young breast cancer patients during endocrine therapy in China’s unique cultural and medical environment.

## Materials and methods

### Study design and patient selection

A convenience sampling method was employed to survey patients who underwent postoperative adjuvant endocrine therapy for breast cancer at Shanghai Ruijin Hospital between January 2021 and July 2021, and who met the inclusion and exclusion criteria. The inclusion criteria were: diagnosed with breast cancer at or before 40 years of age and having a long-term fixed partner; pathologically confirmed primary breast cancer with stage I to IIIA; estrogen receptor and/or progesterone receptor positive; completed comprehensive treatment for breast cancer other than endocrine therapy and took endocrine drugs for at least 6 months; no cognitive impairment, aware of their own condition, and willing to participate in the study. The exclusion criteria were as follows: discontinued endocrine therapy for more than 1 month cognitive impairment or a history of psychiatric illness (meets diagnostic criteria for symptoms of diseases listed in the DSM or ICD; system psychiatric history was determined mainly by referring to medical records); severe disabilities, or mobility impairments [we used Barthel index (10) as an assessment tool for functional independence, and respondents in this survey who scored less than 60 were excluded]. This study was approved by the Ethics Committee of Ruijin Hospital affiliated to Shanghai Jiao Tong University School of Medicine (No. 2019115).

### Data collection

Data collection was conducted during patients’ outpatient medication dispensing, treatment, and follow-up visits. After obtaining informed consent from the patients, eligible participants were invited to complete the survey using either a paper or electronic questionnaire. A total of 220 questionnaires were distributed, and 189 valid questionnaires were returned, resulting in an effective response rate of 85.91%.

This study investigated the potential factors that may influence the sexual function of young breast cancer patients during endocrine therapy, based on Ganz’s breast cancer health concept model (7). The research team identified the relevant measurement variables for this investigation which are listed in Table 1.

**Table 1:** Measuring variables and measurement for patients with breast cancer undergoing endocrine therapy.

Elements	Factors	Measurements
Basic characteristics	Demographic and disease characteristics	Patient information survey
Sexual function	Sexual function and sexual satisfaction	Female sexual function index
Physiologic factor	Treatment-related symptoms	Modified Kupperman scale
Psychologic factor	Body image	Body image scale for breast cancer patients
	Anxiety and depression	Hospital anxiety and depression scale
Family and social support	Social support	Social science research solutions
	Conjugal relation	Revised dyadic adjustment scale

### Patient information survey

The data collection form was designed by the researchers and included two parts: (1) demographic information, education level, marital status, work status, reproductive status, and medical payment method; (2) disease and sexual health-related information, which includes the time of disease diagnosis, disease staging, surgical methods, postoperative adjuvant therapy, information on endocrine therapy, preoperative sexual satisfaction, and the start time of the first sexual activity after surgery.

### Female sexual function index

The Chinese version of the female sexual function index (FSFI) (11) was used to evaluate the sexual function of patients. The FSFI consists of six dimensions: sexual desire, sexual arousal, vaginal lubrication, orgasm, sexual satisfaction, and sexual pain. Higher scores indicate better sexual function. The critical value standard based on the study of Song et al. (12) was used in this study.

### Modified Kupperman scale

The modified Kupperman scale (13) was used to evaluate the occurrence and severity of menopausal symptoms in patients within the last month.

### Body image scale for breast cancer patients

The body image scale for breast cancer patients (BIS) was used to assess the self-image in female breast cancer patients (14).

### Hospital anxiety and depression scale

The hospital anxiety and depression scale-depression subscale (HADS) was used for the evaluation of anxiety and depression symptoms (15).

### Social science research solutions

The social science research solutions (SSRS) compiled by Xiao (16), is used to measure the degree of social support of individuals, which has been widely used in the Chinese population and has good reliability and validity (17). In this study, a score of 37 was used as the threshold, dividing social support into low level (<37 points) and high level (≥37 points).

### Revised dyadic adjustment scale

The revised dyadic adjustment scale (RDAS) is used to evaluate the marital relationship (18). The higher the score, the more harmonious the marital adjustment relationship (19).

### Statistical analyses

Latent class analysis (LCA) was used to construct a latent class model of sexual function characteristics of young breast cancer patients during endocrine therapy. The LCA was conducted using Mplus 8.2. The result of the latent class analysis was used for the identification of FSD high risk patients. Chi-squared test or Fisher’s exact test were performed for the comparison between two groups. Logistic regression was used to analyze the influencing factors of different sexual function characteristics classification in young breast cancer patients during endocrine therapy. SPSS 19.0 for Windows was used for the descriptive statistics and logistic regression analysis. R 3.6.3^1^ and rms package (20) was used for the establishment of nomogram and the analysis of *C*-index and calibration curve. All statistical tests were two-tailed, and a *p*-value of <0.05 was considered statistically significant.

## Results

### Sample description

This study included a total of 189 patients, aged 23 to 40 years, with a median age of 36 years. Of these, 157 patients (83.1%) had completed college education or higher, 180 patients (95.2%) were married, and 162 patients (85.7%) had a history of childbirth. All patients had undergone breast cancer surgery, including 81 cases (42.9%) of mastectomy without breast reconstruction, 84 cases (44.4%) of breast-conserving surgery for breast cancer, and 24 cases (12.7%) of breast reconstruction surgery. 66.1% of patients had received chemotherapy before endocrine therapy, 66.1% had received radiotherapy, and 11.6% had received targeted therapy. The duration of endocrine therapy ranged from 6 months to 10 years, with a median time of 2 years. Among them, 138 patients (73.0%) received ovarian function suppression therapy while receiving endocrine therapy.

In this study of 189 young breast cancer patients, 71 (37.6%) resumed sexual activity for the first time within 6 months after surgery, 61 (32.3%) resumed sexual activity between 6 months and 1 year after surgery, and 57 (30.2%) resumed sexual activity more than 1 year after surgery. 170 patients (89.9%) were satisfied with their sexual life before surgery. Only 54 patients (28.6%) had discussed sexual issues with healthcare providers. 125 patients (66.1%) believed that they did not need sexual health guidance from healthcare providers for their illness. The total incidence of female sexual dysfunction (FSD) in this study was 81.0%, with the following FSFI dimension disorders: low sexual desire (83.1%), sexual arousal disorder (69.3%), orgasmic disorder (68.8%), vaginal lubrication difficulties (57.1%), decreased sexual satisfaction (54.5%), and dyspareunia (54.0%) (Table 2).

**Table 2:** Demographic characteristics, medical information and Sexual health related information of the sample (*n* = 189).

Characteristics	*N* (%)
Age of diagnosis	
≤30	30 (15.9)
>30, ≤35	62 (32.8)
>35, ≤40	97 (51.3)
Educational level	
Junior high school	16 (8.5)
High school	16 (8.5)
College degree	133 (70.4)
Postgraduate or above	24 (12.7)
Marital status	
Married	180 (95.2)
Single	6 (3.2)
Divorced	3 (1.6)
Family structure	
Live alone	4 (2.1)
Living with spouse	23 (12.2)
Living with spouse and children	123 (65.1)
Live with many members	39 (20.6)
Fertility circumstance	
Childless	27 (14.3)
One child	124 (65.5)
Two children or more	38 (20.1)
Work status	
Employed	147 (77.8)
Unemployed	42 (22.2)
Monthly household income *per capita*, CNY	
≤2,500	10 (5.3)
>2, 500, ≤5,000	25 (13.2)
>5,000, ≤10,000	58 (30.7)
>10,000	96 (50.8)
Payment manner of the medical expenses	
Medical insurance	178 (94.2)
Self-paying	11 (5.8)
Medical economy burden	
No	112 (59.3)
Yes	77 (40.7)
Surgical approach	
Mastectomy	81 (42.9)
Breast conserving surgery	84 (44.4)
Mastectomy with breast reconstruction	24 (12.7)
Endocrine therapy	
AIs	91 (48.1)
SERM	98 (51.9)
Radiotherapy	
No	64 (33.9)
Yes	125 (66.1)
Targeted therapy	
No	167 (88.4)
Yes	22 (11.6)
Ovarian function suppression	
No	51 (27.0)
Yes	138 (73.0)
Adjuvant chemotherapy	
No	64 (33.9)
Yes	125 (66.1)
Interval from surgery to first sexual activity	
≤6 months	71 (37.6)
>6 months	118 (62.5)
Satisfaction with sexual activity before breast cancer diagnosis	
No	19 (10.1)
Yes	170 (89.9)
Communication with health care providers about sexual issues	
No	135 (71.4)
Yes	54 (28.6)
The need of medical staff to provide sexual health guidance	
No	125 (66.1)
Yes	64 (33.9)

### Latent class analysis

In this study, latent class analysis was performed to explore the sexual function status of 189 young breast cancer patients during endocrine therapy. To obtain the optimal model, the occurrence of dysfunction in 6 domains of sexual health (desire, arousal, lubrication, orgasm, satisfaction, and pain) based on FSFI were used as indicators (scored as 0/1; 0 indicating no dysfunction, 1 indicating dysfunction) and one to five latent classes were fitted sequentially, with the fit indices for each model assessed (Table 3) using AIC, BIC, Entropy, LMR, and BLRT. The model with one class was analyzed firstly (M1), and as the number of classes increased, the AIC and aBIC values decreased while the entropy values remained stable. The two-class model (M2) had the smallest BIC value. However, considering the unreliable representation of the class that accounted for less than 1% of the total sample (21), the optimal model was determined based on a comprehensive evaluation of the fit indices and clinical significance, which indicated that the two-class model (M2) was the optimal model.

**Table 3:** Fitting results of the latent category model of sexual function status in young breast cancer patients during endocrine therapy.

Model	Loglikelihood	AIC	BIC	aBIC	Entropy	LMR	BLRT	Class probability
M1	432.155	1431.087	1450.537	1431.532	–	–	–	1
M2	87.869	1100.801	1142.943	1101.766	0.934	<0.0001	<0.0001	0.693/0.307
M3	53.710	1080.642	1145.477	1082.127	0.901	0.0002	<0.0001	0.212/0.127/0.661
M4	35.787	1076.719	1164.247	1078.723	0.798	0.0363	<0.0001	0.212/0.312/0.360/0.116
M5	18.686	1073.618	1183.837	1076.141	0.942	0.0016	<0.0001	0.021/0.212/0.249/0.127/0.391

When the model included two classes (M2), the average probability of membership for each class was above 90%, with C1 at 98.8% and C2 at 97.6%, indicating a high level of fit quality and model reliability for M2.

**Figure 1:**
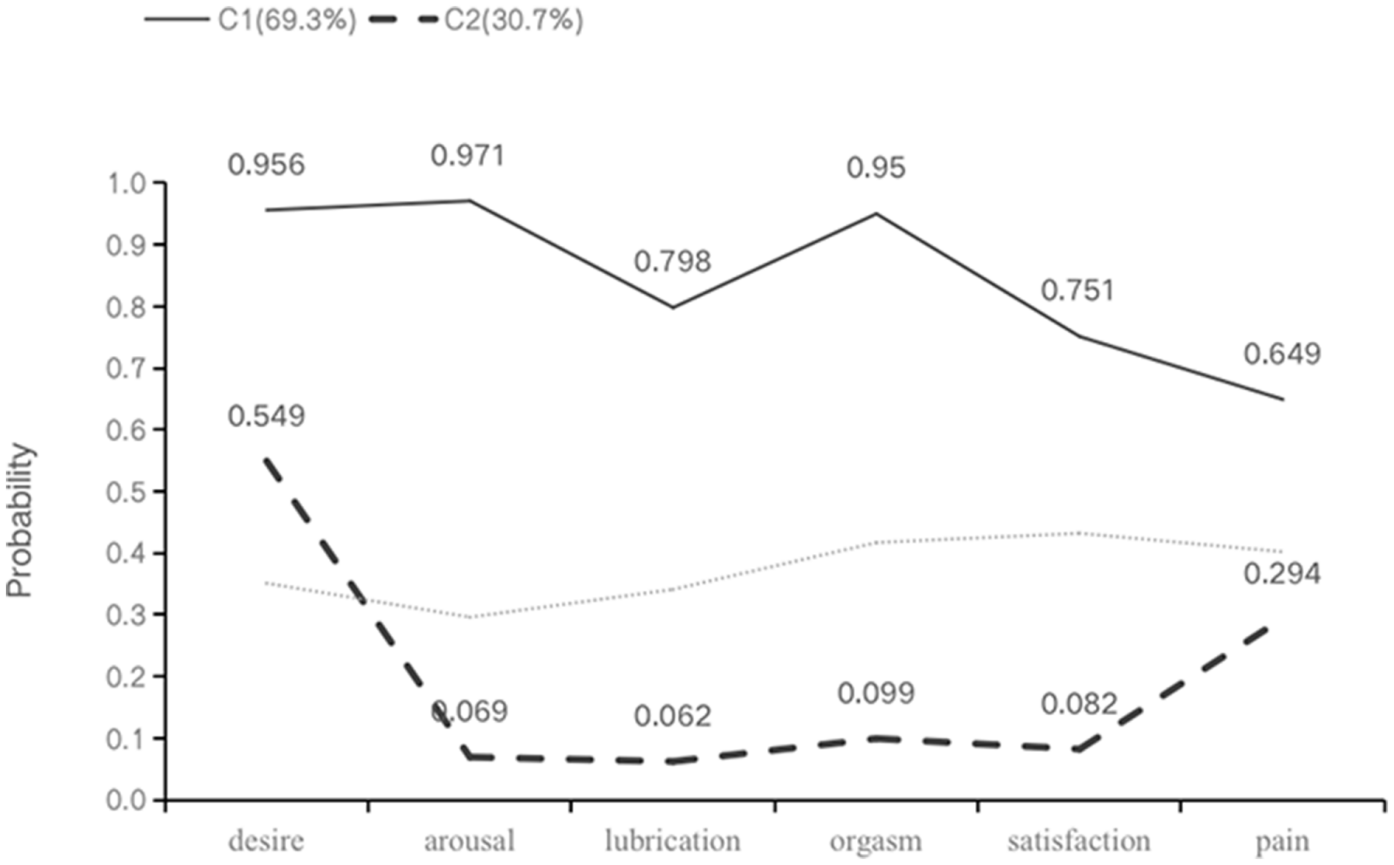
Profiles for two-class LCA model of sexual health. Class 1: C1, high dysfunction-low satisfaction group; class 2: C2, low dysfunction-high satisfaction group.

In the two identified latent classes, C1 had higher rates of sexual dysfunction across all dimensions compared to C2, hence it was labeled as the “high dysfunction-low satisfaction” group with 131 patients (69.3%) falling into this category. Conversely, C2 was labeled as the “low dysfunction-high satisfaction” group relative to C1, with 58 patients (30.7%) belonging to this category (Figure 1). The result of the latent class analysis was used for the identification of FSD high risk patients.

### The logistic regression analysis

In this study, the potential categories of patient sexual function status (C1: low dysfunction-high satisfaction, C2: high dysfunction-low satisfaction) were used as the dependent variable. The variables with *p* < 0.1 in the univariate analysis (marital status, family structure, economic burden, surgical approach, chemotherapy, endocrine therapy, interval from surgery to first sexual activity, satisfaction with sexual activity before breast cancer diagnosis, menopausal symptoms, pre-operative body image, postoperative body image anxiety, depression, social support) and clinically relevant variables (age) were selected as independent variables for binary logistic regression analysis (Table 4). The forward stepwise regression method was used to screen for independent variables. The results showed that the endocrine therapy regimen (AIs/SERM, OR = 2.311, 95% CI 1.101–4.854), postoperative body image (high/low, OR = 2.774, 95% CI 1.329–5.793), medical economic burden (yes/no, OR = 4.385, 95% CI 1.982–9.699), and time of first sexual activity after surgery (late/early，OR = 3.561, 95% CI 1.703–7.444) were the features associated with potential differences in sexual function status among young breast cancer patients during endocrine therapy (*p* < 0.05). However, there were no significant differences in marital status, family structure, surgical approach, satisfaction with sexual activity before breast cancer diagnosis, body image, anxiety, depression, and social support among the potential categories (see Table 5).

**Table 4:** Analysis of different characteristics of young breast cancer patients during endocrine therapy in two identified latent classes (*n*, %).

Factors	“High dysfunction- low satisfaction” group (C1)	“Low dysfunction-high satisfaction” group (C2)	*p*
Age of diagnosis			0.226
≤30	17 (13.0%)	13 (22.4%)	
>30, ≤35	46 (35.1%)	16 (27.6%)	
>35, ≤40	68 (51.9%)	29 (50.0%)	
Educational level			
Junior high school	9 (6.9%)	7 (12.1%)	0.317
High school	12 (9.2%)	4 (6.9%)	
College degree	96 (73.3%)	37 (63.8%)	
Postgraduate or above	14 (10.7%)	10 (17.2%)	
Marital status			
Married	127 (96.9%)	53 (91.4%)	0.011
Single	1 (0.8%)	5 (8.6%)	
Divorced	3 (2.3%)	0 (0.0%)	
Family structure			
Live alone	0 (0.0%)	4 (6.9%)	0.028
Living with spouse	18 (13.7%)	5 (8.6%)	
Living with spouse and children	85 (64.9%)	38 (65.5%)	
Live with many members	28 (21.4%)	11 (19.0%)	
Fertility circumstance			
Childless	17 (13.0%)	10 (17.2%)	0.672
One child	86 (65.6%)	38 (65.5%)	
Two children or more	28 (21.4%)	10 (17.2%)	
Work status			
Employed	98 (74.8%)	49 (84.5%)	0.140
Unemployed	33 (25.2%)	9 (15.5%)	
Monthly household income *per capita*, RMB			
≤2,500	10 (7.6%)	0 (0.0%)	0.190
>2,500, ≤5,000	17 (13.0%)	8 (13.8%)	
>5,000, ≤10,000	40 (30.5%)	18 (31.0%)	
>10,000	64 (48.9%)	32 (55.2%)	
Payment manner of the medical expenses			
Medical insurance	124 (94.7%)	54 (93.1%)	0.739
Self-paying	7 (5.3%)	4 (6.9%)	
Medical economy burden			
No	66 (50.4%)	46 (79.3%)	<0.001
Yes	65 (49.6%)	12 (20.7%)	
Surgical approach			
Mastectomy	53 (40.5%)	28 (48.3%)	0.057
Breast conserving surgery	65 (49.6%)	19 (32.8%)	
Mastectomy with breast reconstruction	13 (9.9%)	11 (19.0%)	
Ovarian function suppression			
No	32 (24.4%)	19 (32.8%)	0.234
Yes	99 (75.6%)	39 (67.2%)	
Chemotherapy			
No	39 (29.8%)	25 (43.1%)	0.074
Yes	92 (70.2%)	33 (56.9%)	
Radiotherapy			
No	41 (31.3%)	23 (39.7%)	0.263
Yes	90 (68.7%)	35 (60.3%)	
Targeted therapy			
No	114 (87.0%)	53 (91.4%)	0.389
Yes	17 (13.0%)	5 (8.6%)	
Endocrine therapy			
AIs	69 (52.7%)	22 (37.9%)	0.061
SERM	62 (47.3%)	36 (62.1%)	
Interval from surgery to first sexual activity			
≤6 months	36 (27.5%)	35 (60.3%)	<0.001
>6 months	95 (72.5%)	23 (39.7%)	
Satisfaction with sexual activity before breast cancer diagnosis			
No	19 (14.5%)	0 (0.0%)	0.002
Yes	112 (85.5%)	58 (100.0%)	
Post-operative body image			
Poor	90 (68.7%)	24 (41.1%)	<0.001
Well	41 (31.3%)	34 (58.6%)	
Communication with health care providers about sexual issues			
No	97 (74.0%)	38 (65.5%)	0.231
Yes	34 (26.0%)	20 (34.5%)	
The need of medical staff to provide sexual health guidance			
No	44 (33.6%)	20 (34.5%)	0.905
Yes	87 (66.4%)	38 (65.5%)	

**Table 5:** Logistic regression analysis of for breast cancer patients undergoing endocrine therapy in different latent class.

Factors	OR	95% CI	*p*
Endocrine therapy, AIs vs. SERM	2.311	1.101–4.854	0.027
Post-operative body image poor vs. well^a^	2.774	1.329–5.793	0.007
Medical economic burden yes vs. no	4.385	1.982–9.699	<0.001
Interval from surgery to first sexual activity late vs. early^a^	3.561	1.703–7.444	0.001

a The postoperative body image score of 5 was taken as the cut-off value; and the delayed first postoperative sex time was defined half a year after breast cancer surgery.

### Prognostic nomogram for female sexual dysfunction

The four independent prognostic factors: Patients received AI combined with OFS treatment (*p* = 0.027), had poor body-image after surgery (*p* = 0.007), beared heavy medical economy burden (*p* < 0.001), and had a delayed recovery of sexual function after surgery (*p* = 0.001) were included in the process of establishment of the nomogram. Patients in class I according to the latent class analysis were identified as patients with female sexual dysfunction.

**Figure 2:**
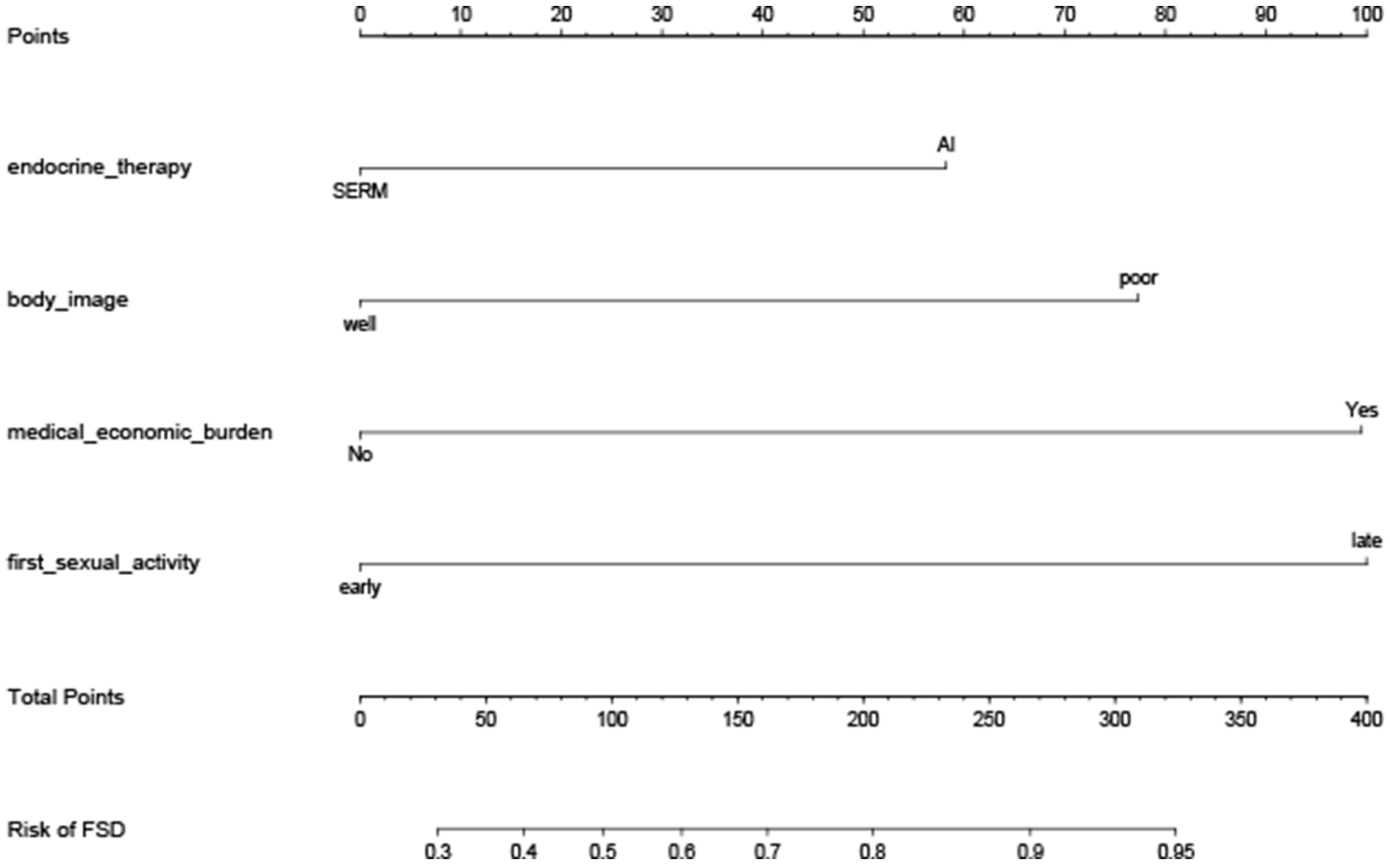
Nomogram for the prediction of FSD. Nomogram for the prediction of FSD in young breast cancer patients during endocrine therapy, with a *C*-index of 0.782.

Prognostic nomograms that integrated the significant independent factors for FSD are established and shown in Figure 2. The *C*-index values of the nomogram in predicting FSD was 0.782.

**Figure 3:**
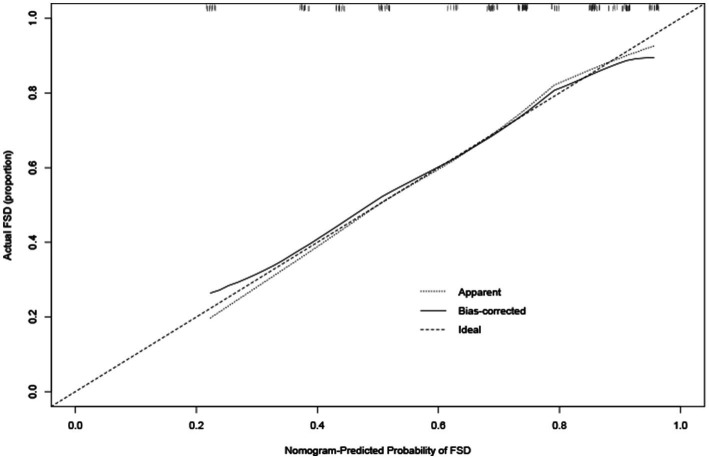
Calibration curve for predicting FSD. Calibration curve for predicting FSD in young breast cancer patients during endocrine therapy. Nomogram-predicted probability of FSD is plotted on the *x*-axis, while actual rate of FSD is plotted on the *y*-axis.

The calibration plot for the probability of FSD showed an optimal agreement between the prediction by the nomogram and actual observations (Figure 3).

## Discussion

In this study, the incidence of female sexual dysfunction (FSD) among young breast cancer patients during endocrine therapy was found to be 81.0% based on the FSFI score. The incidence of each dimension of sexual dysfunction ranged from 54.0% to 83.1%. To better evaluate FSD, latent class analysis was performed. Compared with previous studies on sexual function in breast cancer patients (22), the incidence of FSD in young patients in this study was higher. Lee et al. (23) found that 32% of young breast cancer patients who were sexually active before diagnosis had sexual dysfunction. Zhang’s study of 80 young (≤50 years old) breast cancer patients who received comprehensive treatment found that 70.15% of the patients had sexual dysfunction after treatment (24). The high incidence of FSD in this study may be related to regional and cultural differences in the study population, as well as to the fact that the patients in this study were receiving endocrine therapy. Endocrine therapy can lead to a decrease in estrogen levels, causing sexual desire and arousal disorders, as well as vaginal dryness, decreased elasticity and increased fragility of the vulva and vaginal tissues, resulting in discomfort or pain during sexual intercourse. Other studies have reported that endocrine therapy is a risk factor for sexual desire disorders, vaginal lubrication difficulties and sexual pain in breast cancer patients (25, 26). Domestic studies have also found that patients’ sexual function significantly decreased after 6 months of endocrine therapy, especially in the areas of sexual desire, vaginal lubrication, sexual satisfaction and sexual pain (27).

Moreover, our study found that 71.4% of patients had not discussed their sexual issues with medical staff, and 66.1% of patients believed that they did not need sexual guidance from medical staff. This may be related to the cultural background and social customs in China, where both patients and medical staff often consider sex a private matter. It also reflects that some medical staff do not fully recognize the importance of sexual rehabilitation. Overall, the study findings indicated considerable variability in sexual function among young breast cancer patients undergoing endocrine therapy. The assessment of sexual function using latent class analysis was supported by several statistical fitting indices, and the clinical significance of the identified sexual dysfunction was also demonstrated. Overall, this study highlights the complexity and diversity of sexual function in this patient population and emphasizes the importance of addressing sexual health concerns in the context of breast cancer care. The patients were ultimately divided into a “high disorder-low satisfaction” group and a “low disorder-high satisfaction” group. In both groups, the incidence of decreased sexual desire was higher than the norm, which may be related to the decrease in estrogen levels caused by endocrine therapy.

Currently, there is limited research on the heterogeneity of female sexual dysfunction both domestically and internationally. One study conducted by Yuan et al. (19) on the sexual health characteristics of breast cancer patients had conducted research on it. The latent categories of sexual dysfunction in young breast cancer patients during endocrine therapy identified in this study share some similarities and differences with the research of Yuan et al. (19). Their study classified breast cancer patients based on their sexual activity and analyzed the heterogeneity of their sexual health, identifying five subgroups with different sexual health characteristics. The difference in the classification results may be due to differences in the study population. The subjects included in this study were young patients diagnosed before the age of 40, a population with a strong sexual demand and physiological function, and high sexual activity. In contrast, the study of Yuan et al. (19) included patients with an age range of 25 to 70 years and a median age of 46 years. The results of her heterogeneity analysis of sexually active individuals are consistent with this study, with sexual function and sexual satisfaction being significant classification features of sexual health.

Multivariate analysis showed that the type of endocrine therapy, postoperative body image, medical economic burden, and timing of first sexual activity after surgery were the characteristics associated with latent categories of sexual dysfunction in young breast cancer patients during endocrine therapy.

This study reveals that young breast cancer patients who take AIs are more likely to be classified as “high dysfunction-low satisfaction” group. While endocrine therapy brings therapeutic benefits, it also leads to a series of treatment-related adverse reactions, such as sleep disorders, bone and joint pain, hot flashes, and effects on sexual function. In the ATAC trial comparing the efficacy and safety of AIs and TAM endocrine therapy, researchers evaluated the sexual quality of life of the subjects and found that anastrozole had a greater impact on reduced libido and painful intercourse in patients than TAM (28). In the IES 031 trial, the sexual assessment index showed a lower incidence of vaginal dryness in the TAM group (29). The results of this study are consistent with previous research.

As the use of AIs in young breast cancer patients requires the combination of OFS, the addition of OFS can cause certain toxic side effects, mainly focused on the occurrence of menopausal symptoms, reduced sexual activity, and decreased quality of life. These symptoms are not age-restricted in premenopausal women (30). According to existing literature on the 5 years SOFT and TEXT studies (31), adverse reactions were observed, including significant changes in sexual symptoms from baseline. Patients receiving exemestane + OFS had more severe vaginal dryness symptoms than patients receiving tamoxifen + OFS, and the difference persisted. In terms of libido, patients receiving exemestane + OFS were more likely to experience a decrease in libido than patients receiving tamoxifen + OFS, and the difference increased with the duration of treatment. For sexually active patients at baseline, the risk of sexual arousal difficulties in the exemestane + OFS group was higher than that in the tamoxifen + OFS group. Furthermore, the study results of patients under 35 years old in both trials (32) showed that the use of OFS increased the risk of decreased libido and sexual arousal difficulties, and the risk difference persisted with the increase in treatment time. The AI+OFS group had a higher incidence of vaginal dryness, decreased libido, and sexual arousal difficulties compared to the TAM + OFS group, which may be related to the different mechanisms of action of the drugs. Since there are abundant estrogen receptors in brain tissue, the decrease in estrogen levels and the prevention of estrogen from binding to its receptors may affect the emotional function of the human body (29). Further research is needed to determine whether drug-induced changes in emotional function affect patient sexual function. Therefore, this study suggests that interventions for sexual dysfunction in young breast cancer patients during endocrine therapy should focus on those who use AI in combination with OFS.

The results of this study indicate that patients with poor postoperative body image are more likely to be in the “high dysfunction-low satisfaction” group. Body image reflects the objective perception and subjective evaluation and feelings of patients towards their own body, and is related to characteristics such as emotional expression, appearance recognition, and social interaction. These constituent factors interact in a multidimensional manner in the context of breast cancer (33). The disease itself and its treatment can cause varying degrees of damage to the patient’s body image, which in turn reduces sexual response between spouses. Poor quality of sexual life can also have a negative impact on a patient’s self-image (34). Previous research results have also shown that low self-image scores are significant predictors of low sexual satisfaction, and “feeling unattractive” is the most common reason for not engaging in sexual activity with a partner (35). At the same time, breast cancer patients after surgery are troubled by body image issues for a long time, and the longer the postoperative time, the worse their self-image perception becomes (36). These findings suggest that effective measures to improve body image should be provided to patients as early as possible to improve their sexual function.

The financial burden of medical treatment is often an overlooked factor in studies of this kind. The results of this study show that patients with a heavy economic burden are more likely to be classified in the “high dysfunction-low satisfaction” group. Although more than 90% (94.2%) of the patients in this study were covered by medical insurance, about 40% of them still had varying degrees of economic burden. Some researchers have found that the total medical expenses in the whole society have continued to rise over the past decade, and the average out-of-pocket expenses for patients are also increasing rapidly (37). This phenomenon can provide some explanation for the results of this study. Breast cancer patients undergoing endocrine therapy require regular medication and follow-up, which may result in various indirect economic burdens due to missed work and frequent hospital visits. Economic factors play a significant role in the dynamic changes in patients’ physical and psychological aspects and may be one of the causes of sexual dysfunction in patients.

The research findings indicate that patients with a longer recovery time for sexual activity after surgery are more likely to be classified as belonging to the “high dysfunction-low satisfaction” group. In this study, the proportion of breast cancer patients who resumed sexual activity within 1 month after surgery was 6.9%, which was higher than the reported rate in Anhui province (6.0%) (38), but lower than that in Xi’an (12.64%). Compared to western countries (39), the time for breast cancer patients to resume sexual activity in China is generally later. The appropriate time for sexual activity to resume after surgery should be based on the patient’s recovery status. If there are no complications after surgery, sexual activity can be resumed once the surgical wound has healed. Engaging in sexual activity after breast cancer surgery does not increase the risk of cancer recurrence. On the contrary, sexual activity has been found to enhance emotional intimacy between partners, promote physical and emotional well-being, and potentially contribute to disease recovery. According to the research by Jankowska (40), the first sexual activity after surgery is a turning point for spousal sexual adaptation and adjustment. Communication with spouses, sharing feelings related to breast cancer, and understanding each other’s sexual needs and expectations can promote better sexual satisfaction for both spouses. A follow-up study on young breast cancer patients (41) found that the higher the quality of the first sexual intercourse after surgery, the less emotional distress related to subsequent sexual activity. Researchers have pointed out that breast cancer patients and their spouses will experience a period of sexual recovery after surgery (40). During this period, medical staff should proactively provide relevant guidance on sexual issues to patients, promote effective communication and emotional exchange between spouses, and help patients better cope with sexual problems.

This study has several limitations that need to be addressed. Firstly, its prospective nature and single-center design at Ruijin Hospital affiliated with Shanghai Jiao Tong University School of Medicine may limit the generalizability of the study findings. Although the patient sample included individuals from various regions of the country, the treatment environment was still relatively limited. Therefore, future studies with larger sample sizes and recruitment from multiple centers are needed to validate and enhance the external validity of the findings. Secondly, some patients declined participation in the study due to the sensitive nature of the research topic, which may cause bias. Furthermore, this study focuses on female sexual dysfunction, which is influenced by various factors such as social, physiological, and psychological factors from spouses. However, there is still some missing information about the factors associated with spouses, which may have an impact on the study results. The establishment and validation of the nomogram was based on the data in a single center. Further studies with larger sample sizes are needed to verify the accuracy of the nomogram. Further investigation is needed to explore and clarify the factors that affect sexual function in young breast cancer patients during the endocrine therapy period.

## Conclusion

This study has revealed that among young breast cancer patients undergoing endocrine therapy, the incidence of FSD was high, and FSD was found to be heterogenous. It suggests that healthcare providers should strengthen the evaluation of sexual function status among young breast cancer patients during endocrine therapy, especially those who use AIs, have poor postoperative body image, experience medical economic burden, and delay the recovery of their first sexual intercourse after surgery. Timely targeted support should be provided based on their different characteristics to help breast cancer patients cope with the negative impact of sexual dysfunction and improve their quality of life. The proposed nomogram performed well in predicting FSD for young breast cancer patients undergoing endocrine therapy.

## Data availability statement

The raw data supporting the conclusions of this article will be made available by the authors, without undue reservation.

## Ethics statement

This study was approved by the Ethics Committee of Ruijin Hospital affiliated to Shanghai Jiao Tong University School of Medicine (No. 2019115). Written informed consent from the patient was not required to participate in this study in accordance with the national legislation and the institutional requirements.

## Author contributions

LG and Y-MM contributed equally to this study. LG and NZ designed the research. LG, X-JD, Q-RZ, and QR collected the data. LG and Y-MM analyzed the data and prepared the manuscript. All authors contributed to the article and approved the submitted version.

## Footnotes

^1^ http://www.r-project.org/

## Abbreviations

OFS: Ovarian Function Suppression
FSD: Female Sexual Dysfunction
SERM: Selective Estrogen Receptor Modulator
TAM: Tamoxifen
AIs: Aromatase inhibitors
FSFI: Female Sexual Function Index
BIS: Body Image Scale for Breast Cancer Patients
HADS: Hospital Anxiety and Depression Scale-Depression Subscale
SSRS: Social Science Research Solutions
RDAS: The Revised Dyadic Adjustment Scale
LCA: Latent Class Analysis
AIC: Akaike Information Criterion
BIC: Bayesian Information Criterion
LMR: Lo–Mendell–Rubin
BLRT: Bootstrap Likelihood Ratio Test
